# Exosomes released by human umbilical cord mesenchymal stem cells protect against cisplatin-induced renal oxidative stress and apoptosis *in vivo* and *in vitro*

**DOI:** 10.1186/scrt194

**Published:** 2013-04-25

**Authors:** Ying Zhou, Huitao Xu, Wenrong Xu, Bingying Wang, Huiyi Wu, Yang Tao, Bin Zhang, Mei Wang, Fei Mao, Yongmin Yan, Shuo Gao, Hongbing Gu, Wei Zhu, Hui Qian

**Affiliations:** 1 School of Medical Science and Laboratory Medicine, Jiangsu University, Zhenjiang, Jiangsu, China; 2 Department of Central Laboratory, the First People’s Hospital of Lianyungang, 182 Tongguan Road, Lianyungang, Jiangsu, China; 3 The Affiliated Hospital, Jiangsu University, 228 Jiefang Road, Zhenjiang, Jiangsu, China; 4 Department of Clinical Laboratory Medicine, The Affiliated People’s Hospital of Jiangsu University, Zhenjiang, Jiangsu, China

**Keywords:** Acute kidney injury, Apoptosis, Cisplatin, Exosome, Human umbilical cord mesenchymal stem cells, Oxidative stress, Proliferation

## Abstract

**Introduction:**

Administration of bone marrow mesenchymal stem cells (MSCs) or secreted microvesicles improves recovery from acute kidney injury (AKI). However, the potential roles and mechanisms are not well understood. In the current study, we focused on the protective effect of exosomes derived from human umbilical cord mesenchymal stem cells (hucMSC-ex) on cisplatin-induced nephrotoxicity *in vivo* and *in vitro*.

**Methods:**

We constructed cisplatin-induced AKI rat models. At 24 h after treatment with cisplatin, hucMSC-ex were injected into the kidneys via the renal capsule; human lung fibroblast (HFL-1)-secreted exosomes (HFL-1-ex) were used as controls. All animals were killed at day 5 after administration of cisplatin. Renal function, histological changes, tubular apoptosis and proliferation, and degree of oxidative stress were evaluated. *In vitro*, rat renal tubular epithelial (NRK-52E) cells were treated with or without cisplatin and after 6 h treated with or without exosomes. Cells continued to be cultured for 24 h, and were then harvested for western blotting, apoptosis and detection of degree of oxidative stress.

**Results:**

After administration of cisplatin, there was an increase in blood urea nitrogen (BUN) and creatinine (Cr) levels, apoptosis, necrosis of proximal kidney tubules and formation of abundant tubular protein casts and oxidative stress in rats. Cisplatin-induced AKI rats treated with hucMSC-ex, however, showed a significant reduction in all the above indexes. *In vitro*, treatment with cisplatin alone in NRK-52E cells resulted in an increase in the number of apoptotic cells, oxidative stress and activation of the p38 mitogen-activated protein kinase (p38MAPK) pathway followed by a rise in the expression of caspase 3, and a decrease in cell multiplication, while those results were reversed in the hucMSCs-ex-treated group. Furthermore, it was observed that hucMSC-ex promoted cell proliferation by activation of the extracellular-signal-regulated kinase (ERK)1/2 pathway.

**Conclusions:**

The results in the present study indicate that hucMSC-ex can repair cisplatin-induced AKI in rats and NRK-52E cell injury by ameliorating oxidative stress and cell apoptosis, promoting cell proliferation *in vivo* and *in vitro*. This suggests that hucMSC-ex could be exploited as a potential therapeutic tool in cisplatin-induced nephrotoxicity.

## Introduction

Previous studies have shown that mesenchymal stem cells (MSCs) from different sources, including human bone marrow, cord blood, embryo and fetal membranes can be applied in tissue repair, such as promoting recovery from acute kidney injury (AKI) induced by various causes [1-4]. Similarly, our laboratory has demonstrated that MSCs derived from human umbilical cord and rat bone marrow mesenchymal stem cells can ameliorate ischemia/reperfusion-induced AKI and mouse hepatic injury [5-8]. Moreover, MSCs have been used in clinical trials [9,10]. Recently, microvesicles (MVs) derived from MSCs have been widely applied to experimental research, including AKI. Some studies have demonstrated that microvesicles released by human bone marrow MSCs contribute to repairing AKI induced by glycerol and ischemia-reperfusion [11,12] and improved survival in a lethal model of AKI induced by cisplatin in severe combined immunodeficiency (SCID) mice [13].

Exosomes are microvesicles of multivesicular bodies (MVBs) released from various cells into the extracellular space when intracellular MVBs fuse with the plasma membrane of the cell. Consequently, exosomes are present in cell culture supernatants. Exosomes are composed of a lipid bilayer, and contain proteins, mRNA and miRNA [14,15]. Previous research has indicated that exosomes play a biological role mainly in transmission of proteins, mRNA and miRNA. CD9, CD63 and CD81 have been used as characteristic markers of exosomes [15-17]. In view of these findings, more and more research has been conducted with the aim of investigating the functions of exosomes. The effect of exosomes on AKI, hepatic injury and myocardial ischemia/reperfusion injury has been demonstrated previously [11-13,18,19].

Cisplatin, as an anticancer drug, is often used to induce an animal model of AKI [1,13]. The mechanism of cisplatin renal toxicity is not fully understood; the main reason for AKI occurrence may be renal tubular apoptosis, and the increase of lipid peroxidation and reactive oxygen species (ROS) in the kidney, leading to kidney oxidative damage and renal cell death. At the same time, oxidative damage is one of the triggers for renal cell apoptosis [20-22]. It has also been reported that antioxidant stress pathways are stimulated by mesenchymal stromal cells in renal repair after ischemia-reperfusion injury [23]. Thus, we suggest exosomes derived from MSCs may ameliorate AKI caused by antioxidant stress pathways.

Although exosomes derived from bone marrow mesenchymal stem cells have been successfully applied in improving survival in a lethal model of AKI induced by cisplatin in SCID mice [13], the mechanism and the protective effect of exosomes in an immunocompetent model are unclear. Moreover, the therapeutic potential of exosomes derived from human umbilical cord mesenchymal stem cells (hucMSC-ex) on kidney injury has not been reported so far.

Based on previous studies, the aim of the present study was to investigate the effect of hucMSC-ex on cisplatin-induced AKI. Our findings suggest that exosomes released from hucMSCs could suppress cisplatin-induced AKI and NRK-52E cell injury by improving oxidative stress and cell apoptosis, promoting cell proliferation *in vivo* and *in vitro*.

## Methods

### Isolation and characterization of hucMSCs

All experiment protocols were approved by the medical ethics committee of Jiangsu University (2012258). Fresh umbilical cords were obtained from consenting mothers, and processed within 6 h. HucMSCs were isolated as previously described [24]. All cords were cultured in low glucose Dulbecco’s modified Eagle’s medium (LG-DMEM) containing 10% fetal bovine serum (FBS; Shanghai ExCell Biology, China) and 1% penicillin and streptomycin at 37°C with 5% CO_2_. Medium was replaced every 3 days after initial plating. When confluence reached 80%, the cells were passaged into new flasks for further expansion. As control cells, human lung fibroblasts (HFL-1; Cell Bank, Type Culture Collection Committee, Chinese Academy of Sciences) were cultured with minimal essential medium alpha (MEM-a) containing 15% FBS.

To detect the typical markers of different passages of hucMSCs, flow cytometric analysis was performed using the following fluorescein isothiocyanate (FITC)-conjugated or phycoerythrin (PE)-conjugated antibodies: CD13, CD29, CD44, CD90, CD105, human leukocyte antigen (HLA)-I, CD34, CD45, and HLA-DR (Becton Dickinson, San Jose, CA, USA). Mouse PE-IgG1 and FITC-IgG1 isotypic immunoglobulins were used as isotype controls.

To determine the multidirectional differentiation potential of hucMSCs, the cells of passage 2 were seeded into six-well plates at 1 × 10^5^ cells per well. The next day, the medium was changed to osteogenic medium (0.1 μM dexamethasone, 10 μM β-glycerophosphate, 50 μM ascorbate-phosphate) and adipogenic medium (Cyagen, Guangzhou, China). The medium was replaced every 3 days. After 2 weeks, osteogenic differentiation of hucMSCs was examined by neutrophil alkaline phosphatase (NAP) staining according to the manufacturer’s protocol (SUNBIO, Shanghai, China). At 3 weeks later, adipocytes were stained with Oil-Red-O staining.

### Isolation and characterization of exosomes derived from hucMSCs and HFL-1

HucMSCs and HFL-1 were cultured in serum-free medium. After 48 h, cell culture media were collected and density gradient centrifugations performed. Exosomes were isolated as previously described [18]. Cell supernatants were centrifuged at several times, and then passed through a 0.22-μm filter. Final exosomes were obtained and stored at −70°C. The morphology of the collected exosomes was observed by transmission electron microscopy (FEI Tecnai 12, Philips, The Netherlands). The CD9 (Bioworld, Louis Park, MN, USA), CD63 (Bioworld), and CD81 (Epitomics, Burlingame, USA) molecules, frequently located on the surface of exosomes, were analyzed by western blotting. As described above, after hucMSCs were cultured in media for 48 h, the conditioned media (hucMSC-CM) was collected for the following experiment *in vivo*. Exosome-depleted hucMSC-conditioned media (non-hucMSC-ex) was also gathered for use in the rat model of AKI.

### Rat model of AKI

Adult female Sprague–Dawley rats (weighing 220 ± 20 g) were purchased from the Animal Centre of Chinese Academy of Sciences (Shanghai, China), and housed in a specific pathogen-free animal facility under constant temperature and humidity, and with a 12 h/12 h light/dark cycle with sufficient qualified food and water. All protocols and surgical procedures were approved by the Institutional Animal Care Committee of Jiangsu University.

To evaluate whether the exosomes derived from hucMSCs could repair AKI but not other non-MSC cells or MSC-secreted other cells except MSC, animals were divided into six groups of six rats each, treated as follows. (1) Normal group (no cisplatin treatment). (2) Phosphate-buffered saline (PBS) group: intraperitoneal injection of a single dose of 6 mg/kg cisplatin. Cisplatin (Dezhou Pharmaceutical, Shandong, China) was dissolved in 0.9% saline. After 24 h, both kidneys in one rat received a renal capsule injection of PBS. (3) hucMSC-ex group: 24 h after cisplatin administration, both kidneys in one rat received a renal capsule injection of 200 μg exosomes from hucMSCs. (4) hucMSC-CM group: after cisplatin treatment for 24 h, rats received a renal capsule injection of equal volumes of hucMSC-CM with hucMSC-ex. (5) Non-hucMSC-ex group: after 24 h of cisplatin administration, rats were given a renal capsule injection of exosome-depleted hucMSC-conditioned media. (6) HFL-1-ex group: 24 h after cisplatin treatment, rats received a renal capsule injection of 200 μg exosomes from HFL-1 to both kidneys.

Blood samples were collected from the rats via the tail vein every day and centrifuged at 900 *g* for 15 minutes. Then the serum was separated and stored at −70°C until assayed. Rats were killed at day 5 after administration of cisplatin; both kidneys were immediately excised and cut into two coronal sections each. Two pieces of kidney were fixed in 4% paraformaldehyde at room temperature, the others were stored at −70°C. The blood urea nitrogen (BUN) and creatinine (Cr) levels were determined by a Biochemistry Autoanalyzer (Olympus, Tokyo, Japan).

### Location of hucMSC-ex in AKI kidney

To investigate whether exosomes could incorporate into tubular epithelial cells, immunofluorescence was used. First, we incubated exosomes and CM-Dil dye (Molecular Probes, Eugene, OR, USA) together for 30 minutes at 37°C. The CM-Dil dye-labeled exosomes were injected into the AKI kidneys via the renal capsule, with unlabeled exosomes used as a control. After 24 h, fresh kidney tissues were removed for frozen sectioning. Then, frozen sections were blocked using 5% bovine serum albumin (BSA) for 20 minutes. The sections were then incubated with cytokeratin 19 antibody (Bioworld) at 37°C for 1 h. After washing three times, green fluorescent anti-rabbit antibody was used as secondary antibody. Finally, Hoechst 33342 dye (Sigma Saint Louis, USA) was added to the slices. Cytokeratin 19 antibody was used for staining the cytoplasm of tubular epithelial cells and Hoechst 33342 dye was used for nuclei staining.

### *In vitro* experiments

NRK-52E cells were purchased from Cell Bank, and maintained in DMEM containing 10% newborn calf serum (NBS; Gibco, Grand Island, USA) at 37°C with 5% CO_2_. For *in vitro* treatments, NRK-52E cells were seeded in six-well plates at 1 × 10^5^ cells per well. At approximately 70% confluence, the control and cisplatin groups were grown with or without 5 μM cisplatin for 6 h, then the control and cisplatin groups were changed to fresh medium. In the other two groups, after NRK-52E cells were treated with 5 μM cisplatin for 6 h the culture solutions were changed to 1 mL fresh medium with 160 μg/mL exosomes derived from hucMSCs or HFL-1, respectively. After 24 h, cells were fixed in 4% paraformaldehyde for histologic staining or were collected for protein extraction, and cell suspensions were collected to detect glutathione (GSH) and malondialdehyde (MDA). In order to determine whether hucMSC-ex promote cell proliferation through activation of the extracellular-signal-regulated kinase (ERK)1/2 pathway, cisplatin-treated NRK-52E cells were cultured in fresh medium containing 160 μg/mL hucMSC-ex and 15 μM U0126 (Promega, Wisconsin, USA); 24 h later, cells were collected for protein detection.

### H&E staining

To detect the injury of kidney tubules, the kidneys were fixed in 4% paraformaldehyde (pH 7.4) gradually dehydrated, embedded in paraffin, cut into 4-μM sections and stained with H&E stain.

### Terminal deoxynucleotidyl transferase-mediated dUTP-biotin nick-end labeling (TUNEL) assay

Tissue slices underwent deparaffination and dehydration, then renal tubular cell apoptosis was measured by the TUNEL assay using an *in situ* cell apoptosis detection kit (Boster, Wuhan, China) according to the manufacturer’s instructions.

### Immunohistochemistry

Immunohistochemistry was used for detection of proliferating cell nuclear antigen (PCNA) and the renal oxidative stress product 8-hydroxy-2′-deoxyguanosine (8-OHdG) *in vivo* and *in vitro*. The kidney tissue slices underwent deparaffination and dehydration, and were then immersed in 30% hydrogen peroxide for 10 minutes to block endogenous peroxidase and antigen retrieval for 10 minutes in 0.01 M citrate buffer (pH 6.0) in turn. Then, sections were incubated with PCNA antibody (Bioworld) and 8-OHdG antibody (10 μg/mL, Japan Institute for Control of Aging, Shizuoka, Japan) at 37°C for 1 h. Sections were washed with PBS three times and incubated with biotinylated goat anti-rabbit or anti-mouse IgG (Bostar, Wuhan, China) at 37°C for 20 minutes followed by streptavidin-biotin complex (SABC) for 20 minutes. The antibody binding sites in the tissue slices were visualized with 3,3′-diaminobenzidine (DAB), and counterstained with hematoxylin. *In vitro*, the NRK-52E cells treated in different ways were fixed in 4% paraformaldehyde for 30 minutes. After washing three times with PBS, cells were incubated with 8-OHdG antibody at 37°C for 1 h, and incubated with a secondary antibody for 20 minutes. Cells were visualized with diaminobenzidine substrate and counterstained with hematoxylin. The morphological tissue and cell sections were evaluated by high-power light microscopy examination (Nikon, Tokyo, Japan).

### Mitochondrial membrane potential assay

A mitochondrial membrane potential assay kit with JC-1 staining solution (Beyotime, Nantong, China) was used to detect early apoptosis. After NRK-52E cells were treated in six well plates as described above, the medium was changed for 1 mL fresh medium with 1 mL JC-1 (5 μg/mL) and cells incubated at 37°C for 20 minutes then washed twice with JC-1 staining solution (1 μg/mL). Images were obtained by fluorescent microscopy and analyzed for green and red fluorescence. Mitochondrial membrane potential depolarization was expressed by an increase in the green/red fluorescence intensity ratio.

### Measurement of GSH and MDA

*In vivo*, frozen kidney tissues were thawed, weighed and grinded in homogenate medium (pH 7.4, 0.01 mol/L Tris–HCl, 0.0001 mol/L ethylenediaminetetra-acetic acid (EDTA)-Na_2_, 0.01 mol/L sucrose, 0.8% NaCl solution). Prepared 10% homogenate was centrifuged at 400 *g*, 10 minutes. *In vitro*, cells were collected and ground in PBS. The total protein concentration from kidney tissues or cultured cells was determined using the BCA assay kit (Pierce, USA). Commercial assay kits used to determine GSH and MDA were purchased from Jiancheng Bioengineering Institute (Nanjing, China). All the procedures were performed according to the manufacturer’s instructions.

### Western blotting analysis

Kidney tissues and cells were lysed in radioimmunoprecipitation assay (RIPA) buffer (150 mM NaCl, 1 mM ethylene glycol tetra-acetic acid (EGTA), 0.1% SDS, 1 mM NaF, 1 mM Na_3_VO_4_, 1 mg/mL aprotinin, and 1 mg/mL leupeptin in 10 mM Tris, pH 7.4) containing 1 mM phenylmethanesulfonyl fluoride (PMSF). The protein concentration of each sample was determined using the BCA assay kit. A total of 150 μg protein were electrophoresed on 12% SDS-polyacrylamide gels, which were transferred to polyvinylidene fluoride membranes, blocked with 5% skim milk for 1 h and then blotted against primary antibodies at 4°C overnight. Primary antibodies were as follows: glyceraldehyde 3-phosphate dehydrogenase (GAPDH) (Kangchen Bio-tech, Shanghai, China), B cell lymphoma 2 (Bcl-2) protein (Santa Cruz, Minneapolis, USA), Bcl-2-associated X protein (Bax) (Bioworld), p-ERK (Santa Cruz), ERK (Santa Cruz), phosphorylated p38 mitogen-activated protein kinase (p-p38MAPK) (Santa Cruz) and p38MAPK (Santa Cruz), caspase 3 (Bioworld). After this, the membrane was washed three times with Tris-buffered saline/Tween (TBST) and incubated in goat anti-rabbit or mouse antibodies (Bioworld) for 1 h at 37°C. Western blotting detection was performed using Luminata™ crescendo western horseradish peroxidase (HRP) substrate (Millipore, Billerica, MA, USA).

### Statistical analysis

All data were shown as means ± standard deviation (SD). The statistically significant differences between groups were assessed by analysis of variance (ANOVA) with two-way classification, or ANOVA with Student-Newman-Keuls multicomparison test using Prism software (GraphPad, San Diego, USA). A *P* value of <0.05 was considered significant.

## Results

### Typical features of hucMSCs and hucMSC-ex

**Figure 1:**
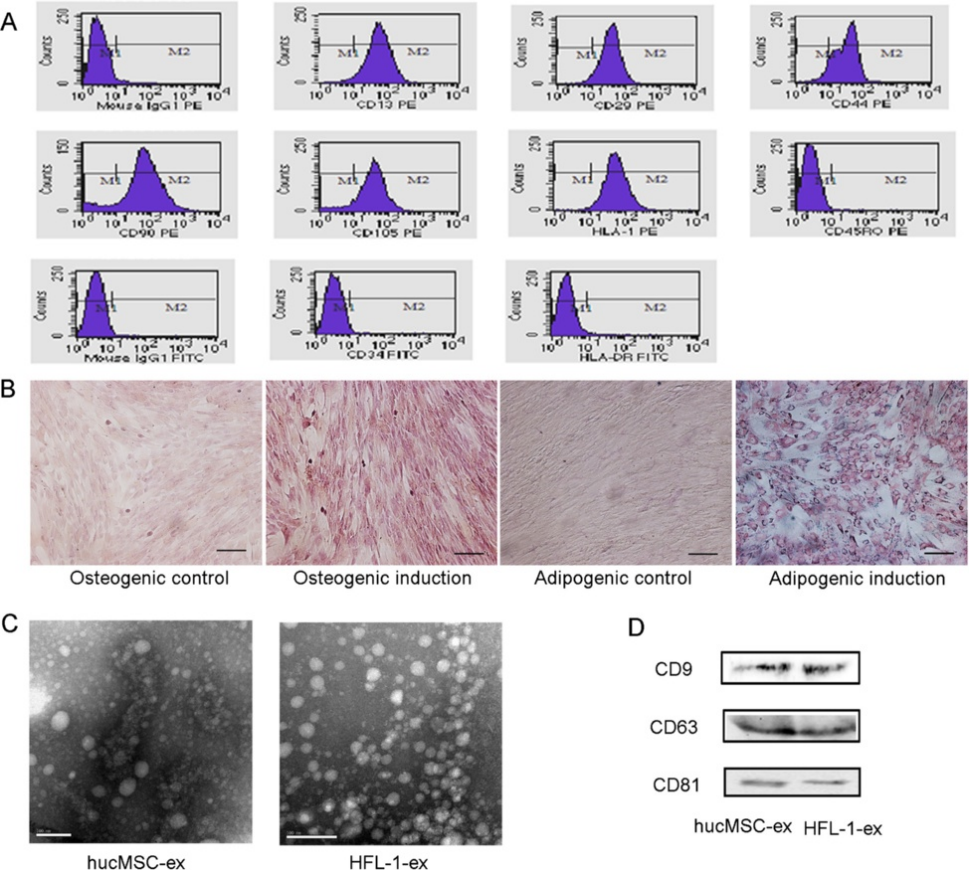
**Characterization of human umbilical cord mesenchymal stem cells (hucMSCs) and hucMSC-derived exosomes (hucMSC-ex). (A)** Flow cytometry analyses of phenotypic markers of hucMSCs; different passages of hucMSCs showed similar results. HucMSCs were positive for CD13, CD29, CD44, CD90, CD105 and human leukocyte antigen (HLA)-I, and negative for CD34, CD45 and HLA-DR. **(B)** Cell lineage induced differentiation. Control cells were grown in regular medium; there were no positive cells in the cell lineage differentiation. Osteogenic differentiation of hucMSCs was shown by neutrophil alkaline phosphatase (NAP) staining; most hucMSCs were alkaline phosphatase positive. Adipogenic differentiation was analyzed by Oil-Red-O staining. After induction, hucMSCs formed numerous Oil-Red-O-positive lipid droplets (shown at original magnification, × 100). Scale bars: 100 μm. **(C)** Representative micrographs of transmission electron microscopy of purified hucMSC-ex and human lung fibroblast secreted exosomes (HFL-1-ex), showing a spheroid shape. The scale bar represents 100 nm. **(D)** Western blotting results indicated the positive expression of CD9, CD63 and CD81 proteins in exosomes derived from hucMSCs and HFL-1 cells.

Fluorescence-activated cell sorting (FACS) analysis demonstrated that hucMSCs expressed high levels of CD13, CD29, CD44, CD90, CD105, and HLA-I, but were negative for CD34, CD45 and HLA-DR (Figure 1A). The hucMSCs we obtained had the typical markers of MSCs. After osteogenic and adipogenic medium induction, some of the hucMSCs became alkaline phosphatase positive and showed numerous Oil-Red-O-positive lipid droplets (Figure 1B, induction). Non-induced cultures did not show spontaneous osteoblast or adipocyte formation (Figure 1B, control). These results suggest that hucMSCs have the ability to differentiate into adipocytes and osteocytes.

Transmission electron microscopy showed the spheroid morphology of exosomes derived from hucMSCs and confirmed their size as 40 to 100 nm. HFL-1-released exosomes showed similar results (Figure 1C). Western blotting analyses indicated that hucMSC-ex and HFL-1-ex expressed exosomal markers such as CD9, CD63 and CD 81 protein (Figure 1D).

### Exosomes derived from hucMSCs improved renal function and ameliorated morphology in cisplatin-induced AKI rats

**Figure 2:**
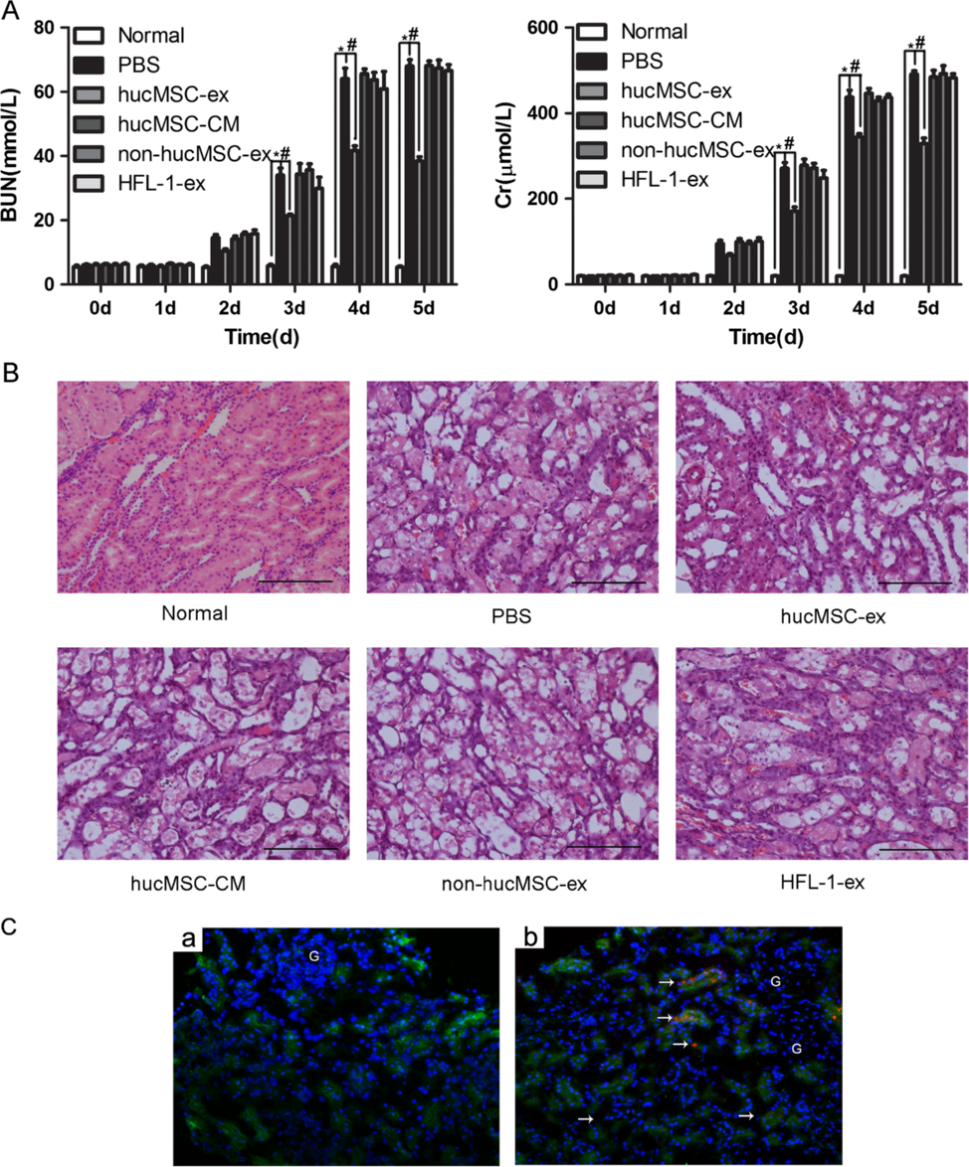
**Exosomes released from human umbilical cord mesenchymal stem cells (hucMSCs) induced an accelerated acute kidney injury (AKI) rat recovery. (A)** Blood urea nitrogen (BUN) and creatinine (Cr) values from 0 days to 5 days. At 24 h after cisplatin administration, rats received a renal capsule injection of 200 μg of exosomes from hucMSCs per kidney. Analysis of variance (ANOVA) with two-way classification was performed: *P <0.001, phosphate-buffered saline (PBS) group vs normal group; ^#^*P* <0.001, hucMSC-ex group vs PBS group. Results showed that BUN and Cr levels were significantly lower in the hucMSC-ex group than in the PBS, hucMSC-CM, non-hucMSC-ex and human lung fibroblast secreted exosomes (HFL-1-ex) groups from 3 days to 5 days. There were no marked changes in the PBS, hucMSC-CM, non-hucMSC-ex and HFL-1-ex groups. **(B)** Representative micrographs of renal histology at day 5 in the four groups. There were numerous examples of necrosis of the proximal epithelium and tubular protein casts in the PBS, hucMSC-CM, non-hucMSC-ex and HFL-1-ex groups, but this was not the case in the hucMSC-ex group. Original magnification, × 200. Scale bars: 100 μm. Group, n = 6. **(C)** Incorporation of hucMSC-ex in tubular epithelial cells by renal capsule injection: **(a)** unlabeled hucMSC-ex was injected into the kidney, no red fluorescence was detected; **(b)** red fluorescence (white arrows) was observed in the tubular epithelial cells after injection of CM-Dil dye-labeled hucMSC-ex at 24 h. Tubular epithelial cell cytoplasm and nuclei were stained green and blue, respectively. Original magnification × 200.

At 2 days after cisplatin injection, we observed a significant rise in BUN and Cr levels in the PBS group and these values increased with time. Renal function was clearly improved from day 3 to day 5 after the rats were treated with hucMSC exosomes (*P <*0.001). However, the BUN and Cr levels in the hucMSC-CM, non-hucMSC-ex and HFL-1-ex groups had no notable changes compared to the PBS group (Figure 2A). Lesions were observed in rats with cisplatin injection at day 5 by H&E staining of kidney tissue slices. The slices showed that numerous necrotic areas of the proximal epithelium had appeared and abundant tubular protein casts had formed in the PBS, hucMSC-CM, non-hucMSC-ex and HFL-1-ex groups. However, the tubular lesions markedly decreased in the hucMSC-ex group (Figure 2B). After CM-Dil-labeled hucMSC-ex was injected at 24 h, red fluorescence was detected in the tubular epithelial cells (Figure 2C(b)). The red fluorescence was not observed in unlabeled hucMSC-ex administration kidney samples (Figure 2C(a)).

### HucMSC-ex inhibited cisplatin-induced renal tubular apoptosis and improved renal cell proliferation in rats

**Figure 3:**
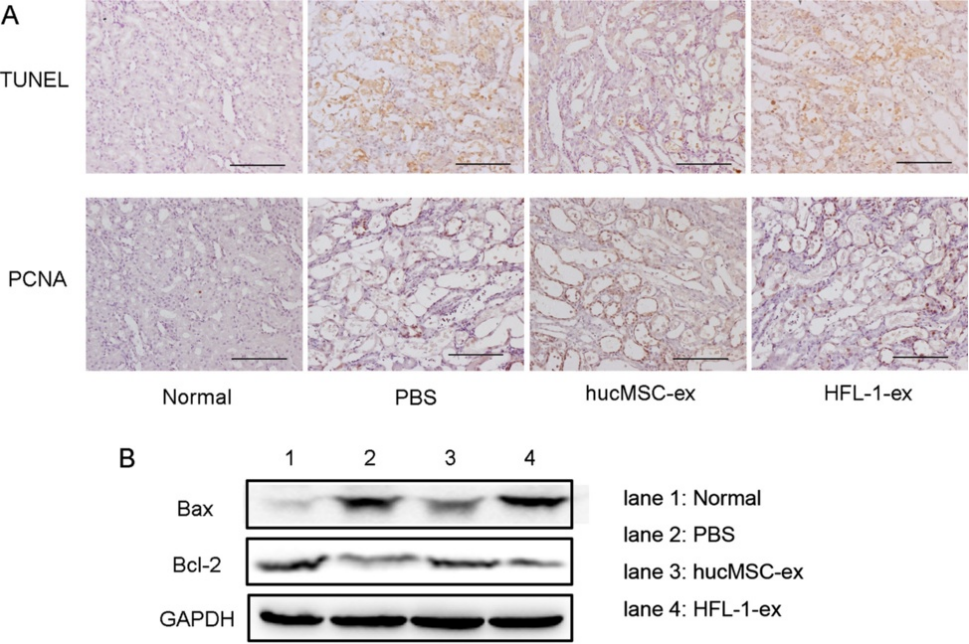
**Human umbilical cord mesenchymal stem cell (hucMSC) exosomes suppressed renal cell apoptosis and promoted renal cell proliferation in cisplatin-induced acute kidney injury (AKI) rats. (A)** Representative images of terminal deoxynucleotidyl transferase-mediated dUTP-biotin nick-end labeling (TUNEL)-positive cells notably increased in the phosphate-buffered saline (PBS) and human lung fibroblast secreted exosomes (HFL-1-ex) groups compared to the hucMSC-ex group, while proliferating cell nuclear antigen (PCNA)-positive cells were clearly more numerous in the hucMSCs-ex group than the PBS and HFL-1-ex groups. Original magnification, × 200, Scale bars: 100 μm. **(B)** Western blot analysis of B cell lymphoma 2 (Bcl-2) protein and Bcl-2-associated X protein levels in the kidney tissues showed that Bax expression increased while the expression of Bcl-2 was reduced in the PBS and HFL-1-ex groups. This phenomenon was reversed in hucMSC-ex-treated group.

As shown in Figure 3A, renal tubular cell apoptosis appeared in cisplatin-induced AKI rats, and hucMSC-ex repressed apoptotic action and enhanced tubular cell proliferation in the cisplatin-induced AKI model. The number of TUNEL-positive cells significantly increased after cisplatin treatment compared to the normal group, while the kidneys of hucMSC-ex-treated rats subjected to cisplatin exhibited less TUNEL-positive cells. Furthermore, AKI rats treated with hucMSC-ex displayed a marked increase in PCNA-positive cells compared to untreated AKI rats (Figure 3A). The expression of Bax and Bcl-2 protein were observed by western blotting in kidney tissues. The level of Bax was evidently raised in the PBS group compared to the normal group. After treatment with hucMSC-ex, its expression was reduced. Bcl-2 expression in the hucMSC-ex group was also reversed (Figure 3B). The HFL-1-ex group showed similar results to the PBS group.

### HucMSC-ex depressed cisplatin-induced renal oxidative stress *in vivo* and *in vitro*

**Figure 4:**
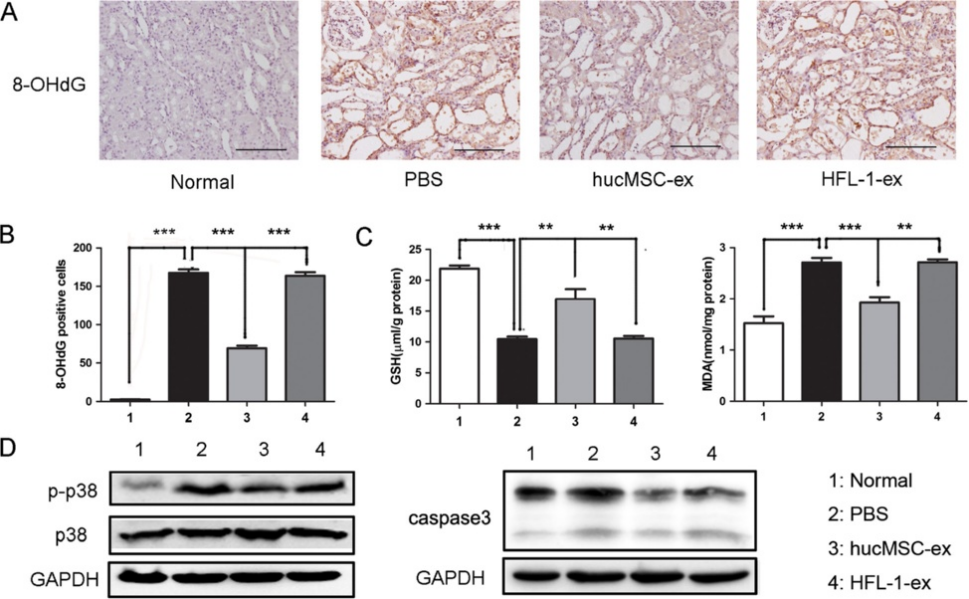
**Human umbilical cord mesenchymal stem cell (hucMSC) exosomes attenuated tubular oxidative damage in cisplatin-induced acute kidney injury (AKI) rats. (A)** Immunohistochemistry was used to detect the oxidative stress marker 8-hydroxy-2′-deoxyguanosine (8-OHdG). The number of 8-OHdG-positive cells decreased in hucMSC-ex-treated rats compared to untreated rats. Original magnification, × 200, Scale bars: 100 μm. **(B)** Quantitative analysis of 8-OHdG-positive cells indicated that hucMSC-ex reduced cisplatin-induced kidney oxidative damage. Analysis of variance (ANOVA) was performed with the Student-Newman-Keuls multicomparison test. ***P <0.001. **(C)** Glutathione (GSH) and malondialdehyde (MDA) levels were examined in kidney tissue homogenates with the respective detection kits (see Methods). GSH expression was reduced, while MDA content increased in the phosphate-buffered saline (PBS) and human lung fibroblast secreted exosomes (HFL-1-ex) groups, but this phenomenon was reversed in the hucMSC-ex group. ANOVA was performed with the Student-Newman-Keuls multicomparison test. **P <0.01, ***P <0.001*.***(D)** Western blot analysis showed that the expression of p-p38 and caspase 3 was higher in the PBS and HFL-1-ex group, while it was suppressed by hucMSC-ex. It indicated that hucMSC-ex inhibited activation of p38 mitogen-activated protein kinase (p38MAPK) by cisplatin-induced oxidative stress and suppressed p38MAPK induced caspase 3 expression, thereby decreasing cell apoptosis.

**Figure 5:**
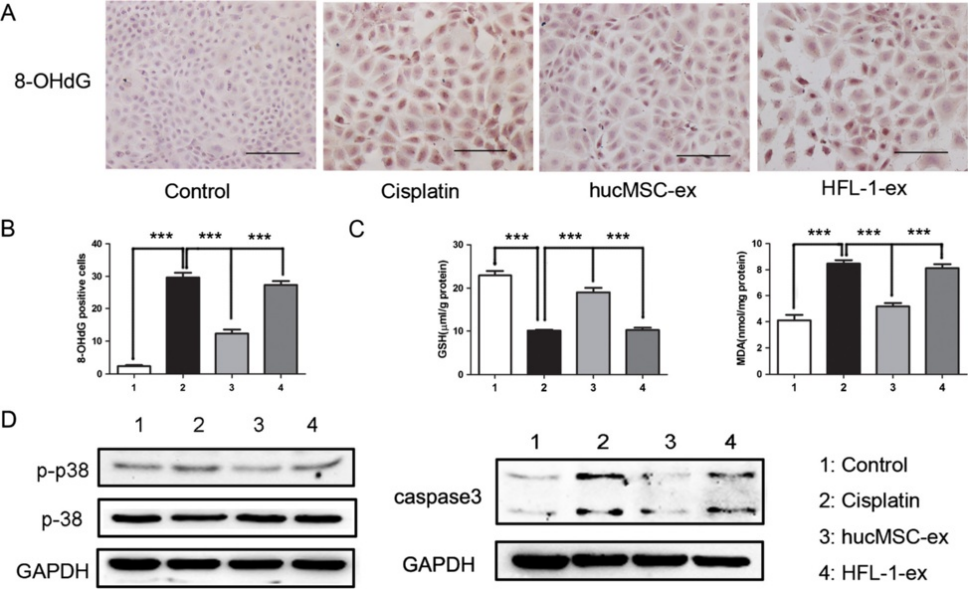
**Human umbilical cord mesenchymal stem cell (hucMSC) exosomes depressed cisplatin-induced NRK-52E cell oxidative damage *in vitro*. (A)** Immunohistochemistry was used to measure levels of the oxidative stress marker 8-hydroxy-2′-deoxyguanosine (8-OHdG). The number of 8-OHdG-positive cells was raised in the cisplatin and human lung fibroblast secreted exosomes (HFL-1-ex) groups compared to the control group, which was reduced by hucMSCs-ex. Original magnification, × 200, Scale bars: 100 μm. **(B)** Quantitative analysis of 8-OHdG-positive cells indicated that hucMSC-ex inhibited cisplatin-induced NRK-52E cell oxidative damage. Analysis of variance (ANOVA) was performed with the Student-Newman-Keuls multicomparison test. ***P <0.001. **(C)** Glutathione (GSH) and malondialdehyde (MDA) levels were examined in cell homogenates. GSH levels decreased and MDA levels increased in the cisplatin and HFL-1-ex groups; this was reversed in hucMSC-ex. ANOVA was performed with the Student-Newman-Keuls multicomparison test. **P <0.01, ***P <0.001. **(D)** Western blot results showed that p-p38 and caspase 3 expression were lower in the hucMSC-ex group than in the cisplatin and HFL-1-ex groups, which demonstrated that hucMSC-ex suppressed activation of p38 mitogen-activated protein kinase (p38MAPK) by cisplatin-induced oxidative stress and depressed the expression of caspase 3, which is increased by p38MAPK.

The cisplatin-induced rat model of AKI is accompanied by oxidative stress. In the cisplatin-treated group *in vivo* and *in vitro*, the oxidative stress product 8-OHdG notably increased, which decreased significantly in the hucMSC-ex-treated group (Figures 4A and 5A). 8-OHdG-positive cells were counted in ten consecutive fields in the sections. This showed that the level of 8-OHdG was higher in the cisplatin and HFL-1-ex groups than in the hucMSC-ex group (*P <*0*.*001) (Figures 4B and 5B). The GSH levels were reduced, and the levels of MDA remarkably enhanced in the cisplatin-treated group compared to the normal group. When comparing the hucMSC-ex-treated group with the PBS and cisplatin groups, the levels of GSH (*P <*0*.*01) were clearly increased, while the MDA levels (*P <*0*.*001) decreased (Figures 4C and 5C). Western blot results suggested that cisplatin-induced oxidative stress activated the p38MAPK pathway, which was suppressed by hucMSC-ex. The activated p38MAPK pathway could lead to caspase 3 expression, but it was inhibited after hucMSC-ex treatment. The HFL-1-ex group had no significant changes compared to the PBS and cisplatin groups *in vivo* and *in vitro* (Figures 4D and 5D).

### HucMSC-ex suppressed cisplatin-induced apoptosis and promoted proliferation in NRK-52E cells

**Figure 6:**
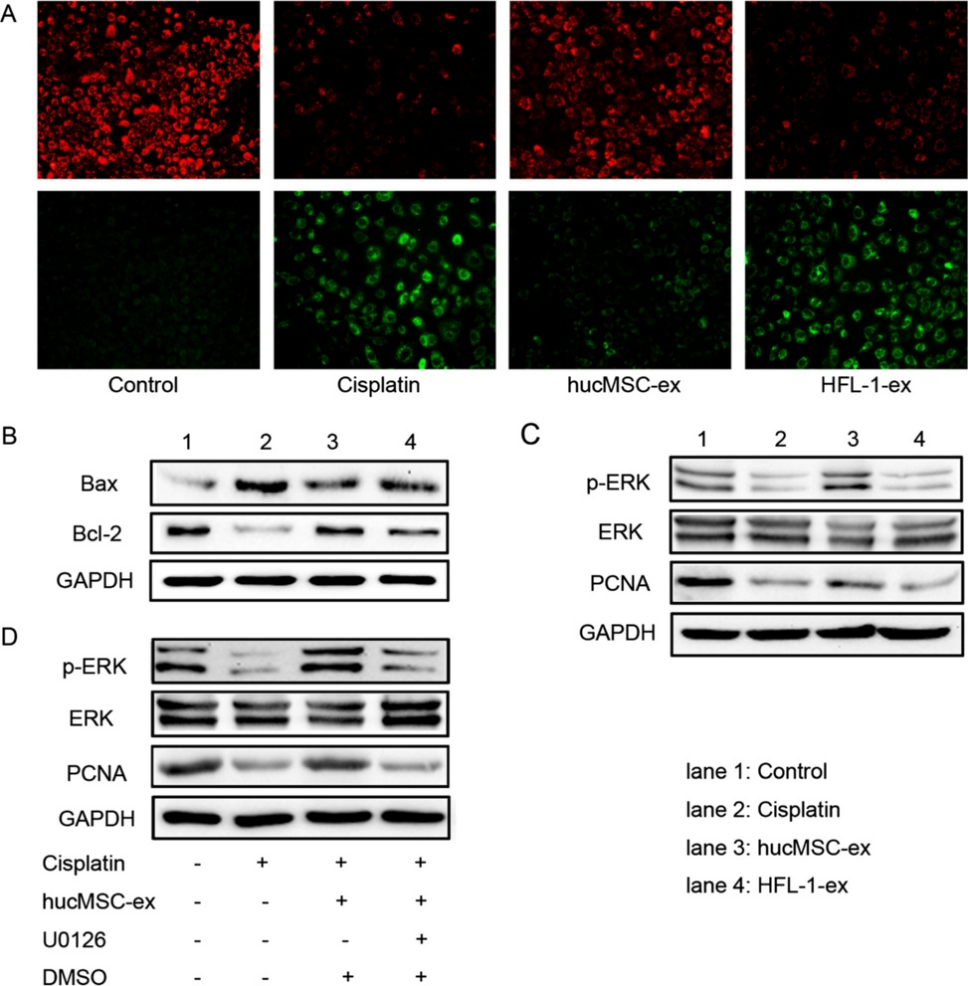
**Exosomes released from human umbilical cord mesenchymal stem cells (hucMSCs) inhibited cisplatin-induced apoptosis and promoted proliferation in NRK-52E cells. (A)** Mitochondrial membrane potentials were analyzed to evaluate cell early apoptosis. The ratio of green to red fluorescence was higher in the cisplatin and human lung fibroblast secreted exosomes (HFL-1-ex) groups than untreated NRK-52E cells, suggesting early apoptosis of cells. The ratio was reversed after treating with hucMSC-ex in cisplatin-induced NRK-52E cells. Original magnification, × 200. **(B)** Western blot analysis of B cell lymphoma 2 (Bcl-2) protein and Bcl-2-associated X protein (Bax) levels in NRK-52E cells: Bax expression was raised in the phosphate-buffered saline (PBS) and HFL-1-ex groups compared to the control group, and this was reversed in the hucMSC-ex group. The expression pattern for Bcl-2 was also reversed in the hucMSC-ex group. **(C)** Western blot results indicated increased phosphorylated extracellular-signal-regulated kinase (p-ERK) expression in the hucMSC-ex group compared to the cisplatin and HFL-1-ex groups, correlating with raised proliferating cell nuclear antigen (PCNA) expression. **(D)** HucMSC-ex and U0126 were added into the medium of cisplatin-treated NRK-52E cells at the same time; p-ERK and PCNA expression were suppressed, as shown by western blot. This indicates that hucMSC-ex promoted injured NRK-52E cell proliferation by activation of the ERK1/2 pathway.

Treatment with cisplatin and cisplatin plus HFL-1-ex resulted in an increase in green fluorescence and a reduction in red fluorescence compared to NRK-52E cells without cisplatin treatment (control group); the ratio of green to red fluorescence ratio increased correlating with an increase in mitochondrial membrane potential. It indicated that NRK-52E cells generated early apoptosis by treatment of cisplatin. After treatment with hucMSC-ex, the ratio of green to red fluorescence was reduced, suggesting the number of apoptosis cells decreased (Figure 6A). The expression of apoptosis-associated proteins Bax and Bcl-2 was observed by western blotting. The level of Bax was seemingly higher in the cisplatin group than the control group, but after treatment with hucMSC-ex its expression decreased. In the cisplatin group, the expression of Bcl-2 was lower than the control group. However, it was increased by treatment of hucMSC-ex (Figure 6B). Western blot analyses also showed that PCNA and phosphorylated-ERK expression decreased markedly in the cisplatin group, which indicated that the cell proliferation ability was obviously weakened. The incubation of cisplatin-treated NRK-52E cells with hucMSC-ex caused higher level of PCNA and p-ERK. There was no evident alteration in the HFL-1-ex group compared to the cisplatin group (Figure 6C).

In order to determine the mechanism of hucMSC-ex action in promoting cell proliferation, we added mitogen-activated protein kinase kinase (MEK) inhibitor to hucMSC-ex-treated NRK-52E cells. Western blot results showed that p-ERK was markedly increased in the hucMSC-ex group compared to the cisplatin group; this phenomenon was inhibited by U0126 and the PCNA expression was also suppressed subsequently (Figure 6D).

## Discussion

Our laboratory has previously demonstrated that MSCs derived from human umbilical cords could repair ischemia/reperfusion-induced AKI [5,6], but the exact mechanism is unclear. Therefore, we considered whether the effect of hucMSCs on AKI rats was via a paracrine secretion mechanism. At the same time, recent studies have showed that nephrotoxicity induced by cisplatin is mainly caused by oxidative stress damage inducing renal tubular epithelial cell apoptosis [22,25]. Based on these findings, in the present study we demonstrated that exosomes released by hucMSCs can ameliorate cisplatin-induced acute kidney injury by antioxidation and antiapoptosis actions and promotion of cell proliferation.

Exosomes are 40 to 100 nm in size and spheroid in shape, and are secreted by various cells. They contain both mRNA and microRNA, and can mediate communication between cells [14-16]. It has been reported that CD9, CD63, and CD81 are located frequently on the surface of exosomes [17]. The exosomes we obtained from hucMSCs and HFL-1 had the same characteristics: transmission electron microscopy showed a spheroid morphology and confirmed their size of 40 to 100 nm, and western blotting analyses showed they were positive CD9, CD63, and CD81 expression. Therefore, they were used in the experiment.

In the present study, we observed that exosomes could become incorporated into the injured tubular epithelial cells, thus accelerating the functional and morphologic recovery of AKI induced by cisplatin. After administration of hucMSC-ex, the level of BUN and Cr was significantly limited, and necrosis of proximal epithelium and abundant tubular protein casts were reduced compared to the PBS, hucMSC-CM, non-hucMSC-ex and HFL-ex groups. It was suggested that cisplatin-induced AKI was associated with a rise in the proapoptotic protein Bax and a reduction in the antiapoptotic protein Bcl-2 [26]. In our study, the hucMSC-ex group had an evident decrease in Bax compared to the cisplatin alone treatment group, while Bcl-2 protein increased after treating with hucMSC-ex *in vivo* and *in vitro*. In addition, the number of apoptotic cells was markedly increased in the PBS group, while it was reduced by hucMSC-ex *in vivo*. Mitochondrial membrane potential detection was used for cell early apoptosis; the ratio of green to red fluorescence increased correlating with an increase in mitochondrial depolarization, which means that cells are exposed to apoptosis [27]. We demonstrated that the ratio of green to red fluorescence was higher in cisplatin-treated NRK-52E cells than controls, and it was reduced by hucMSC-ex.

MSC-ex can promote kidney tubular cell proliferation [11], but the mechanism is unclear. In this study, we found that hucMSC-ex could activate ERK1/2 to promote damaged cell proliferation, and subsequently renal injury repair. Expression of PCNA in the group treated with hucMSC-ex significantly increased compared to the PBS and cisplatin groups *in vivo* and *in vitro*. In order to prove for definite cell proliferation was by activation of the ERK1/2 pathway, *in vitro*, the MEK inhibitor U0126 and hucMSC-ex were added to cisplatin-induced NRK-52E cells at the same time. Western blot analyses indicated that after administration of U0126, p-ERK was suppressed and the expression of PCNA was also depressed. In addition, we also found that hucMSCs-ex could reverse oxidative stress to repair the kidney injury.

Cisplatin-induced oxidative stress was considered to be the main reason for AKI occurrence. Oxidative stress leads to lipid peroxidation and thereby formation of the harmful product MDA [28], and induces DNA oxidative damage accordingly via generation of 8-OHdG [29]. Meanwhile, the activity of antioxidant defense enzymes is decreased [30], which leads to cell damage. These cause the change in the oxidative stress markers: antioxidant enzyme GSH levels were reduced, MDA expression increased and 8-OHdG generated. In the current study, it was observed that hucMSC-released exosomes had an antioxidant role. MDA and 8-OHdG markedly increased in the cisplatin-treated group *in vivo* and *in vitro*, while it was reduced by hucMSC-ex. GSH content significantly increased in the hucMSC-ex-treated group compared to the cisplatin-treated group *in vivo* and *in vitro*. It has already been reported that cisplatin-induced toxicity is closely associated with ROS generated by cisplatin, which leads to oxidative stress [31]. This activates MAPK kinases 3, 4 and 6, which ultimately leads to the activation of p38MAPK. The activated p38MAPK can increase caspase 3 expression, thus leading to cells apoptosis [20,32,33]. In this study, we indicate that the cisplatin-treated group had higher p-p38 and caspase 3 levels compared to the control group, while this was significantly reduced after treatment with hucMSC-ex compared to the cisplatin-treated group *in vivo* and *in vitro*. These results further support the involvement of oxidative stress in cisplatin-induced nephrotoxicity. It is a foundation for clinical treatment of cisplatin-induced acute renal injury.

The precise mechanism of how hucMSC-derived exosomes repair cisplatin-induced AKI is still unclear, but it can be concluded from the results of the present study that hucMSC-ex could be resistant to cisplatin-induced kidney oxidative stress, renal cell apoptosis and promote cell proliferation, thus ameliorating cisplatin-induced acute kidney injury. However, in the above results there were no marked changes when comparing the cisplatin-treated group *in vivo* and *in vitro* to the HFL-1-ex group.

## Conclusions

As is well known, cisplatin treatment results in pronounced kidney oxidative stress and renal cell apoptosis *in vivo* and *in vitro*. Based on the present study, the following conclusions may be drawn: firstly, hucMSC-excreted exosomes can resist cisplatin-induced oxidative stress by reducing formation of some harmful products (8-OHdG, MDA), increasing GSH levels and suppressing activation of the p38MAPK pathway. Secondly, treatment with hucMSC-ex reduced the apoptosis of cells caused by oxidative stress and other reasons, and promoted cell proliferation through activation of ERK1/2. In summary, hucMSC-ex could ameliorate cisplatin-induced nephrotoxicity and could be exploited as a new therapeutic tool for regenerative medicine.

## Abbreviations

AKI: Acute kidney injury; BUN: Blood urea nitrogen; Cr: Creatinine; FITC: Fluorescein isothiocyanate; GSH: Glutathione; HFL-1: Human lung fibroblasts; hucMSC: Human umbilical cord mesenchymal stem cells; 8-OHdG: 8-hydroxy-2′-deoxyguanosine; MDA: Malondialdehyde; PCNA: Proliferating cell nuclear antigen; PE: Phycoerythrin; PMSF: Phenylmethanesulfonyl fluoride.

## Competing interests

The authors declare that they have no competing interests.

## Authors’ contributions

YZ, HX, HQ and WX: designed and performed research, data analysis and manuscript writing; BW and BZ: contributed to acquisition of data; HW, HBG and YT: analysis of data and revision of it critically; FM, YY and SG: analysis and interpretation of data; MW and WZ: designed the study and drafted the manuscript. All authors read and approved the final manuscript for publication.
